# Analysis of risk factors for poor prognosis after endovascular treatment of tandem lesions in acute ischemic stroke

**DOI:** 10.3389/fneur.2025.1628374

**Published:** 2025-09-18

**Authors:** Yifan Zhang, Cheng Cao, Xin Huang, Xinmin Zhou

**Affiliations:** 1 The Jiangyin Clinical College of Xuzhou Medical University, Jiangyin, China; 2 Neurosurgery Department, Jiangyin People’s Hospital, Jiangyin, China

**Keywords:** tandem lesions, endovascular procedures, prognosis, mechanical thrombectomy, acute ischemic stroke

## Abstract

**Aims/background:**

In China, the incidence of acute ischemic stroke (AIS) has been rising annually, accounting for 60–70% of all stroke cases. To explore the risk factors leading to poor prognosis after endovascular treatment in patients with acute ischemic stroke tandem lesions after endovascular treatment.

**Methods:**

A retrospective analysis was conducted on the clinical data of patients with tandem lesions who underwent endovascular therapy at Jiangyin People’s Hospital affiliated with Xuzhou Medical University, from July 2018 to August 2023. Post-treatment revascularization was assessed using the modified Thrombolysis in Cerebral Infarction (mTICI) grading system, with grades 3 and 2b indicating good recanalization and grades 2a and below indicating poor recanalization. Patient prognosis at 90 days post-procedure was evaluated using the modified Rankin Scale (mRS), with scores of 0–2 classified as good prognosis and 3–6 as poor prognosis. The age, admission National Institutes of Health Stroke Scale score (NIHSS), gender, hypertension, diabetes mellitus, coronary artery disease, smoking, atrial fibrillation, carotid artery stenting, revascularization, site of tandem lesion (anterior or posterior circulation), and onset-recanalization time (minutes) of the enrolled patients were subjected to univariate analysis. Univariate analysis was performed, followed by multivariate logistic regression for variables with *p* < 0.1.

**Results:**

A total of 75 patients were included, of whom 32 had good 90-day outcomes and 43 had poor outcomes. Compared to the good outcome group, patients with poor outcomes were older, had higher NIHSS scores at admission, and were less likely to achieve good recanalization (all *p* < 0.05). Multivariate analysis identified older age, higher NIHSS score, and poor recanalization as independent predictors of poor prognosis. The area under the ROC curve for the NIHSS score was 0.735 (*p* = 0.001), indicating moderate predictive value.

**Conclusion:**

Advanced age, elevated NIHSS score on admission, and suboptimal recanalization are independently associated with poor 90-day outcomes following endovascular treatment for tandem lesions. The NIHSS score may aid in early risk stratification.

## Introduction

Acute ischemic stroke (AIS) is a leading cause of mortality and long-term disability, and its incidence continues to rise in China as the population ages. It now accounts for approximately 80% of all stroke cases nationally (1). Among patients with large vessel occlusion (LVO), approximately 10 to 20% present with tandem lesions (TLs), which involve both extracranial internal carotid artery stenosis or occlusion and simultaneous intracranial arterial occlusion (2). These patients are often more critically ill, require more complex procedures, and face a higher risk of poor functional outcomes compared to those with isolated LVO.

Endovascular therapy (EVT) has become the standard treatment for LVO strokes, and its use in TLs has expanded over the past decade. Several studies suggest that EVT is safe and effective in selected TL cases, particularly those involving the anterior circulation (3). However, some past studies have shown comparable reperfusion outcomes for anterior circulation TLs when mechanical thrombectomy is combined with emergent carotid artery stenting (1, 4). Thus, EVT for TLs remains classified as Class IIa, Level B evidence in current guidelines, reflecting ongoing uncertainty and variability in clinical practice.

This study aimed to evaluate clinical predictors of 90-day functional outcome in patients with AIS due to tandem lesions treated with EVT. Specifically, we examined the association between age, admission NIHSS score, and angiographic reperfusion status and post-treatment prognosis in a real-world cohort that included both anterior and posterior circulation TLs.

## Methods

### Study population

We conducted a retrospective analysis of consecutive patients with acute TLs who underwent endovascular treatment at Jiangyin People’s Hospital, affiliated with Xuzhou Medical University, between August 2018 and August 2023. All cases were reviewed according to a predefined eligibility framework based on national diagnostic criteria and contemporary clinical practice guidelines.

Patients were eligible for inclusion: (1) first-ever acute ischemic stroke (AIS) patients meeting diagnostic criteria per the Chinese Guidelines for Diagnosis and Treatment of Acute Ischemic Stroke 2019; (2) onset-to-puncture (OTP) time ≤24 h; (3) absence of intracranial hemorrhage confirmed by initial cranial computed tomography (CT) and, where applicable, supplementary imaging; (4) confirmed extracranial carotid pathology (complete occlusion or severe stenosis) with concurrent intracranial arterial occlusion by CT angiography (CTA), MR angiography (MRA), or digital subtraction angiography (DSA); (5) all patients receiving endovascular therapy (EVT), including mechanical thrombectomy (MT) or stent placement; (6) age ≥18 years; (7) National Institutes of Health Stroke Scale (NIHSS) score ≥6; (8) signed informed consent from patients/legal representatives with complete clinical documentation.

Patients with following were excluded from the study: (1) OTP ≥ 24 h or wake-up stroke with potential OTP > 24 h; (2) pre-stroke modified Rankin Scale (mRS) > 2, or history of cerebral infarction/hemorrhage; (3) concurrent major organ failure (cardiac/hepatic/renal); (4) active malignancy or neoplastic disease; (5) non-adherence to prescribed medications, incomplete rehabilitation, or recurrent cerebral infarction within 90 days post-discharge.

This study was conducted under the principles of the Declaration of Helsinki. Ethical approval was obtained from Jiangyin People’s Hospital. Written informed consent was obtained from all participants before the study (Ethical Review Number: JYPH-EC-2022-045). This study was approved by the Ethics Committee of Jiangyin Clinical College of Xuzhou Medical University (2015–021), and all participants provided informed consent.

### Endovascular treatment

The endovascular management of cerebrovascular tandem lesions typically follows these standardized steps: (1) Vascular access establishment: Percutaneous femoral artery puncture is performed, followed by sheath insertion and guide catheter navigation to the proximal segment of the target lesion. (2) Priority intracranial revascularization: Mechanical thrombectomy (e.g., stent retriever) or aspiration catheter is employed to remove intracranial thrombi, achieving successful blood flow restoration (mTICI ≥ 2b). (3) Extracranial lesion management: Balloon angioplasty is conducted for extracranial stenosis/occlusion (e.g., carotid artery), with stent implantation if required. Intraprocedural tirofiban (a glycoprotein IIb/IIIa inhibitor) is administered to prevent acute thrombosis. (4) Postoperative management: Blood pressure is tightly controlled (systolic <140 mmHg), and dual antiplatelet therapy (aspirin + clopidogrel) is initiated. Cranial CT is repeated within 24 h to exclude hemorrhage.

### Data collection

Baseline demographic and clinical data were retrospectively collected from the medical records of all eligible patients. Variables included age, sex, smoking status, history of hypertension, diabetes mellitus, coronary artery disease, and atrial fibrillation. Procedural and radiological data included lesion location (classified as anterior or posterior circulation), admission National Institutes of Health Stroke Scale (NIHSS) score, onset-to-recanalization time, recanalization success based on modified Thrombolysis in Cerebral Infarction (mTICI) grading (5), and whether carotid stenting was performed. Functional outcome at 90 days was assessed using the modified Rankin Scale (mRS) (6). A good prognosis was defined as an mRS score of 0–2, while a poor prognosis was defined as an mRS score of 3–6. Recanalization was classified as good (mTICI ≥ 2b) or poor (mTICI ≤ 2a).

### Statistical analysis

Continuous variables are expressed as mean (SD) in the case of normal distribution or medians (interquartile range) otherwise. Categorical variables are expressed as *n* (%). Normality of distributions was assessed using the Shapiro–Wilk test. Group differences evaluated using the chi-square test for the categorical variables and Mann–Whitney *U*-test for the continuous variables.

Univariable analyses were used to identify factors potentially associated with poor functional outcome at 90 days, defined as mRS scores of 3 to 6. For univariable logistic regression analyses, we adjusted for multiple comparisons using the Benjamini–Hochberg procedure to control the false discovery rate. Variables with a *p*-value less than 0.1 or deemed clinically relevant were entered into a multivariable logistic regression model to identify independent predictors. Model stability was considered by limiting the number of predictors relative to the outcome events. Adjusted odds ratios (aOR) and 95% confidence intervals (CI) were reported for each covariate. Model performance was further evaluated using receiver operating characteristic (ROC) curve analysis, and the area under the curve (AUC) was calculated to assess discriminative capacity. Complete case analysis was performed, as the primary outcome had no missing data. All statistical tests were two-sided, and a *p*-value < 0.05 was considered statistically significant. Analyses were conducted using SPSS version 26.0 (IBM Corp, Armonk, NY, United States).

## Results

**Figure 1:**
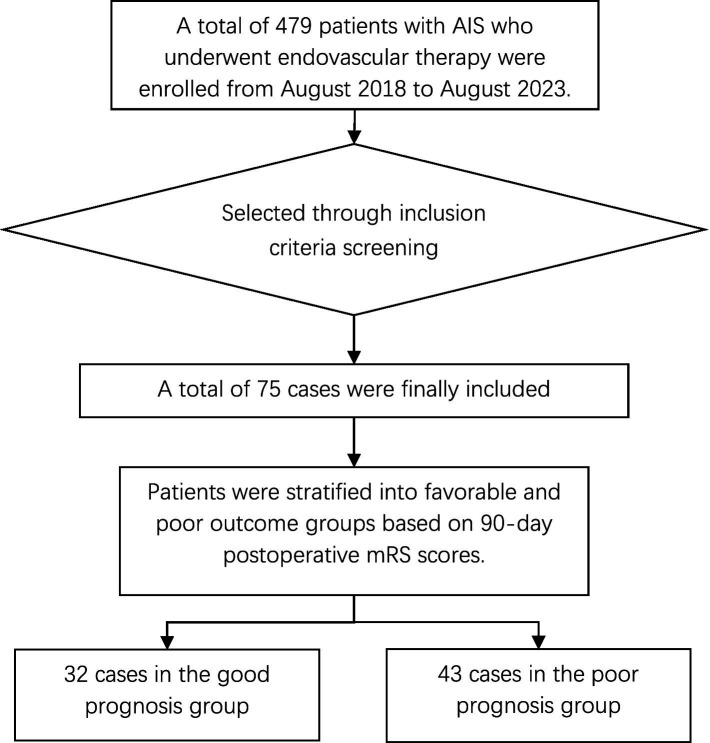
Selection of individuals included in the study.

Among the 75 patients included in the study (Figure 1), the median age was 62 years (IQR: 53–70), and 81.3% (n = 61) were male. As shown in Table 1, patients with poor functional outcomes at 90 days (mRS 3–6) were older (median age 67 vs. 58 years; *p* = 0.009) and had more severe neurological deficits at admission (median NIHSS 16 vs. 12; *p* = 0.001) compared to those with good outcomes. Although distributions of hypertension, diabetes, coronary artery disease, smoking, atrial fibrillation, and lesion location (anterior vs. posterior circulation) were similar between groups, successful recanalization was less frequent in the poor outcome group [33 of 43 (76.7%) vs. of 32 (96.9%); *p* = 0.035]. Other variables, including stent placement and admission blood count parameters, did not differ significantly between outcome groups. Age, NIHSS score, and onset-recanalization time did not follow a normal distribution [Shapiro–Wilk = 0.953, 0.940, 0.895; *p* = 0.028, 0.008, 0.001 < 0.05].

**Table 1:** Univariate analysis of different prognoses in patients with acute tandem lesions.

Parameters	Good prognosis group (*n* = 32)	Poor prognosis group (*n* = 43)	χ^2^(Z)	*p*
Age	58 (47.3, 66.0)	67 (58.0, 72.0)	2.626	0.009
Sex			1.475	0.225
Male	24 (75%)	37 (86%)		
Female	8 (25%)	6 (14%)		
Disease				
Hypertension	19 (59.38%)	31 (72.09%)	1.334	0.248
Diabetes	5 (15.63%)	8 (18.60%)	0.114	0.736
Coronary heart disease	1 (3.13%)	4 (9.30)	0.351	0.553
Smoke	5 (15.63%)	8 (18.60%)	0.114	0.736
Atrial fibrillation	3 (9.38%)	6 (13.95%)	0.060	0.807
Place of disease			1.579	0.209
Anterior cerebral circulation	27 (84%)	31 (72%)		
Posterior cerebral circulation	5 (16%)	12 (28%)		
Onset-recirculation time (min)	394 (335, 452.5)	425 (380, 540)	2.134	0.174
Admission NIHSS score	12 (9, 16)	16 (12, 24)	3.468	0.001
Stent implantation (yes)	5 (15.63%)	10 (23.26%)	2.146	0.143
Revascularization good (mTICI)	31 (96.88%)	33 (76.74%)	4.441	0.035
Admission blood count				
Neutrophils	8.42 (7.02, 9.69)	8.85 (6.81, 11.16)	3.215	0.520
Lymphocyte	1.15 (0.87, 1.47)	0.88 (0.81, 1.33)	1.321	0.247
Platelet	217 (168.50, 266.50)	217 (168.50, 266.50)	0.865	0.623
Monocyte	0.57 (0.38, 0.68)	0.53 (0.38, 0.68)	0.314	0.465

Count data were expressed as median (Q1, Q3). Categorical variables were expressed as *n* (%).

Count data were expressed as median (Q1, Q3). Categorical variables were expressed as *n* (%).

In the multivariable logistic regression (Table 2), older age (OR 1.07, 95% CI 1.02–1.14, *p* = 0.015), higher admission NIHSS score (OR 1.20, 95% CI 1.08–1.38, *p* = 0.003), poor recanalization status (OR 0.04, 95% CI 0.00–0.47, *p* = 0.024), and higher neutrophil count (OR 1.69, 95% CI 1.20–2.55, *p* = 0.006) were independently associated with poor 90-day outcomes. Univariable associations are presented in Supplementary Table S1. After applying the Benjamini–Hochberg correction (Supplementary Table S1), only the NIHSS score remained statistically significant (adjusted *p* = 0.032). Age (adjusted *p* = 0.069) and neutrophil count (adjusted *p* = 0.056) showed trends toward significance.

**Table 2:** Multivariable logistic regression results for poor 90-day outcome.

Variable	aOR (95% CI)*	*p*-value
Age	1.07 (1.02‚ 1.14)	0.015
NIHSS	1.20 (1.08‚ 1.38)	0.003
mTICI score	0.04 (0.00–0.47)	0.024
Neutrophil count	1.69 (1.20‚ 2.55)	0.006

*aOR, adjusted odds ratio.

**Figure 2:**
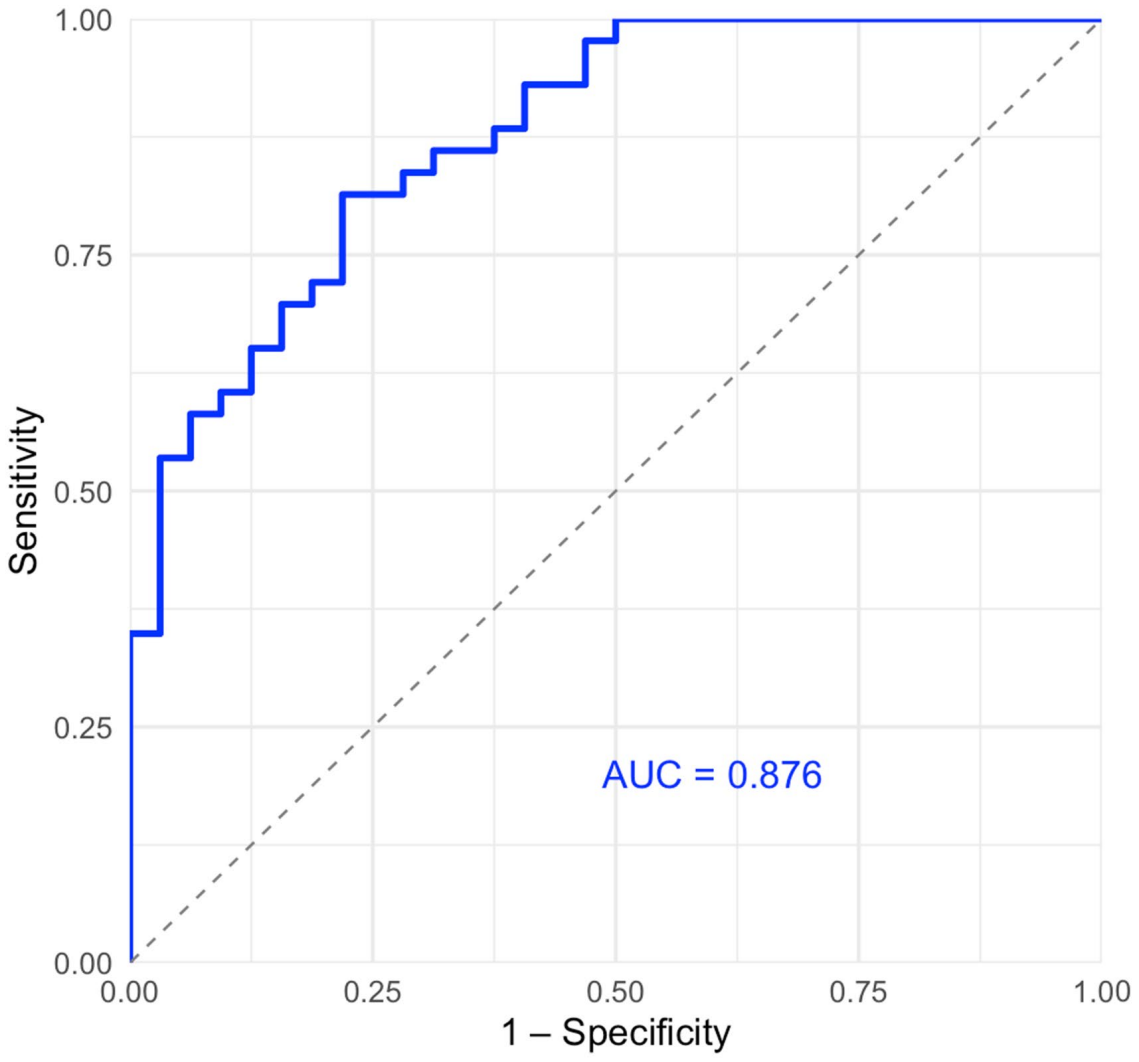
ROC curve plot.

ROC curves were plotted in Figure 2. The model, incorporating age, admission NIHSS score, mTICI grade, and neutrophil count, demonstrated good predictive accuracy for poor functional outcomes at 90 days, with an area under the curve of 0.876.

## Discussion

This study investigated clinical factors associated with poor 90-day outcomes among patients with acute ischemic stroke due to tandem lesions undergoing endovascular treatment. We found that older age, higher baseline NIHSS score, poor recanalization (as measured by mTICI), and elevated neutrophil counts were significantly associated with poor functional outcomes.

This study demonstrated that advanced age is an independent risk factor for poor prognosis in patients. As age increases, patients exhibit reduced tolerance to surgical interventions, prolonged postoperative recovery times, and a higher incidence of complications, all of which negatively impact clinical outcomes (7). Similarly, the baseline NIHSS score, which reflects initial stroke severity, demonstrated strong predictive value. This is consistent with previous research that has established a clear association between higher admission NIHSS scores and long-term disability in ischemic stroke (8). This study reveals the possibility that systemic inflammation, particularly inflammation reflected by elevated neutrophil counts, is an important factor influencing stroke prognosis (9–12). This finding suggests that inflammatory responses may lead to secondary brain injury or impair microvascular reperfusion. Recent literature has emphasized the role of neutrophil-mediated injury in exacerbating ischemic injury and limiting the effectiveness of revascularization therapy in acute stroke patients (13).

Our finding that vascular recanalization status is a critical determinant of prognosis in patients with TLs is consistent with previous studies (14, 15). In this study, the overall successful recanalization rate was 80% (60/75). Among those with successful recanalization, nearly half of the patients achieved a favorable functional outcome, while the majority of patients with poor recanalization remained functionally dependent. These observations align with the consensus in recent studies suggesting that prompt and effective restoration of cerebral blood flow is critical for recovery (16). Despite the consensus on the benefits of recanalization, the optimal procedural strategy for tandem occlusions remains a subject of ongoing debate (17). For example, Cohen et al. (18) reported that for posterior circulation tandem lesions, prioritizing the opening of distal occlusions through non-occluded pathways is a preferred approach. Another method involves addressing the distal occlusion first, followed by the proximal lesion (19). Each approach has inherent advantages and limitations. The proximal-to-distal strategy allows for early stabilization of access and reduces the risk of distal embolization but may delay intracranial reperfusion, potentially leading to larger infarct volumes. In contrast, the distal-to-proximal method facilitates faster reperfusion of critical intracranial vessels and may limit ischemic injury, but is technically demanding and can increase the risk of complications during proximal stenting or balloon angioplasty. In our institution, the distal-to-proximal approach, prioritizing the opening of distal occlusions to promptly improve distal blood flow and reduce infarct size, was applied more. The specific surgical strategy should be tailored to individual patient characteristics to optimize therapeutic outcomes.

**Figure 3:**
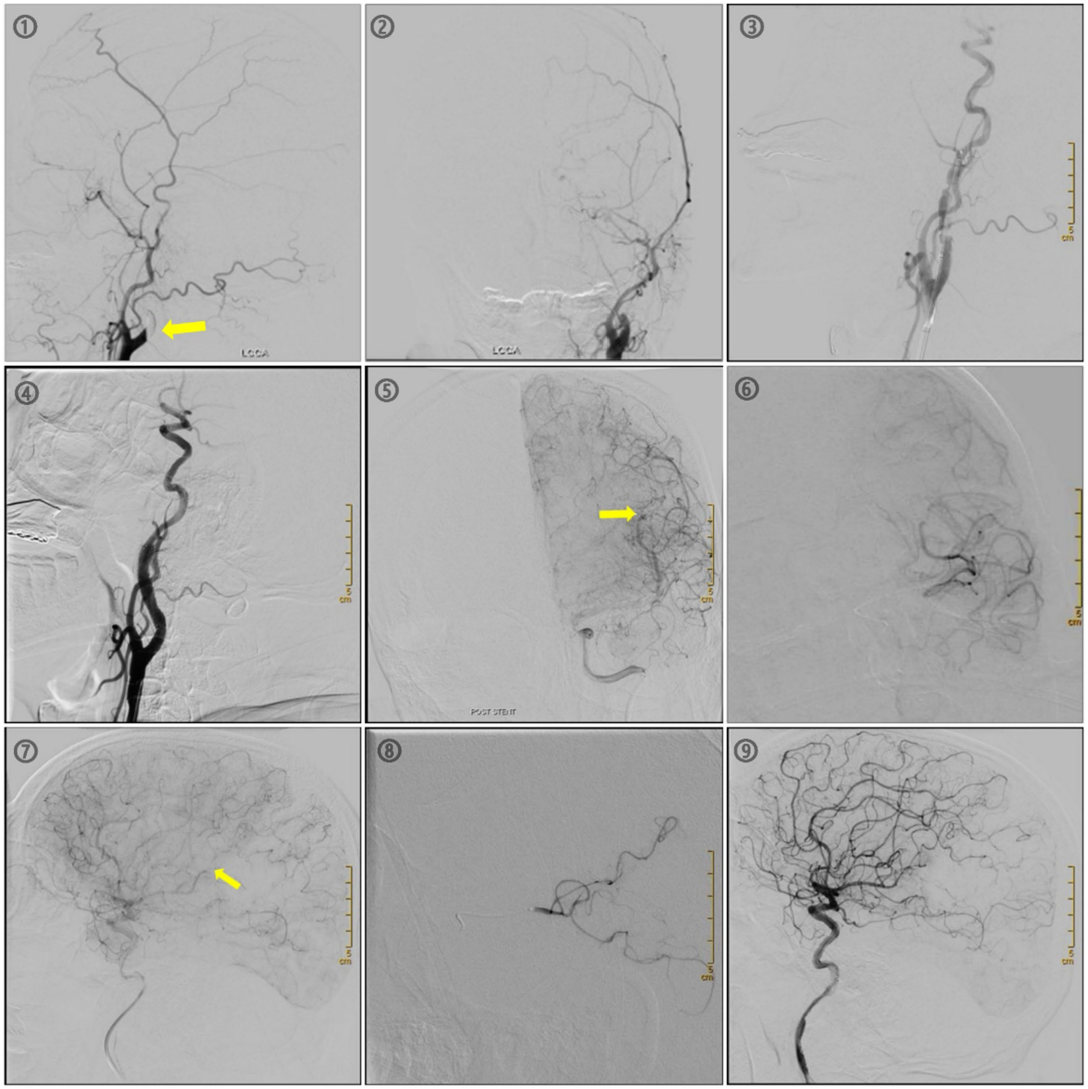
Therapeutic images of a patient treated with endovascular therapy for a tandem lesion of the left common carotid and the M1 of middle cerebral artery.

①DSA imaging shows occlusion of the left common carotid artery (LCCA)② LCCA orthotopic contrast ③ Stent positioning after performing LCCA balloon dilatation ④LCCA contrast after 7 × 40 stent release ⑤ Occlusion of the M1 segment of the middle cerebral artery was seen on intracranial imaging ⑥M1 image⑦ Lateral M1image⑧ Microcatheter passes through M1 segment after M2 imaging shows patency after M2 segment ⑨. Final image after removing the thrombosis (Figure 3).

Tandem lesions often involve atherosclerotic narrowing or occlusion of the extracranial internal carotid artery, making stent implantation a frequent necessity. The decision to place a stent in the acute setting introduces important therapeutic challenges, especially regarding the use of antiplatelet therapy. To prevent in-stent thrombosis, early administration of antiplatelet agents is typically required. However, this practice raises safety concerns, particularly in patients with large infarcts or a high risk of hemorrhagic transformation. Some studies reported that among patients with tandem lesions treated with stent implantation, early anticoagulation with tirofiban showed increased the likelihood of rapid recanalization (20–22). Another study further showed that low-dose micro pumping of tirofiban after endovascular therapy improves the prognosis of patients with TLs without increasing the risk of symptomatic intracranial hemorrhage (23). In our cohort, 15 patients received tirofiban infusion following stent placement. Although the sample size was limited, a substantial proportion of these patients achieved good functional outcomes, which is consistent with previously published findings.

In this study, lesion location, whether in the anterior or posterior circulation, was not significantly associated with 90-day functional outcomes. However, existing literature suggests that posterior circulation tandem lesions (with a favorable prognosis rate of 29.41%, 5/17) are more severe and associated with poorer outcomes compared to anterior circulation tandem lesions (favorable prognosis rate of 46.55%, 27/58). Several anatomical and physiological factors may account for this trend. The posterior cerebral circulation benefits from a network of robust collateral pathways, including connections between the posterior cerebral artery, middle cerebral artery, and anterior cerebral artery through leptomeningeal anastomoses, along with the posterior communicating arteries that form part of the Circle of Willis. These collateral routes can help sustain perfusion during proximal arterial occlusion, delay the progression of ischemic injury, and potentially extend the therapeutic window for intervention. In contrast, anterior circulation tandem lesions, particularly those involving extracranial internal carotid artery occlusion, often lack extensive collateral support before the vessel enters the cranial cavity. This anatomical limitation renders the affected territory more dependent on rapid and complete revascularization to prevent irreversible ischemic damage (24–27). Our study suggested that incomplete recanalization might independently associated with poor outcomes regardless of lesion location. This supports the conclusion that timely and successful reperfusion remains a central determinant of prognosis in patients with tandem lesions. Endovascular therapy, by improving recanalization rates, continues to represent an effective and essential strategy for managing this high-risk population (28, 29, 30).

Several limitations should be acknowledged. First, the study was retrospective and conducted at a single center, which may introduce selection bias and limit the generalizability of the results. Second, the sample size was relatively small, with only 75 patients included, and a particularly low number of cases involving posterior circulation tandem lesions, reflecting the rarity and lower intervention rates in this subgroup. Third, external validation was not performed, and the lack of a prospective design limits the ability to infer causality. Finally, some relevant clinical or imaging factors—such as infarct core volume or collateral status—were not available and may further inform outcome stratification. Future prospective, multicenter studies with larger and more diverse cohorts are needed to validate these associations, improve the robustness of the evidence, and refine risk stratification in this complex stroke population.

In summary, older age, higher admission NIHSS scores, and poor recanalization were independently associated with unfavorable outcomes following endovascular treatment in patients with acute ischemic stroke due to tandem lesions. Among these factors, the NIHSS score on admission demonstrated strong discriminative value, although its sensitivity was suboptimal despite good specificity at a threshold of 18. These findings highlight the clinical importance of initial stroke severity and successful reperfusion in shaping post-treatment outcomes.

## Data Availability

The raw data supporting the conclusions of this article will be made available by the authors, without undue reservation.
