# Correction: Analysis of risk factors for poor prognosis after endovascular treatment of tandem lesions in acute ischemic stroke

**DOI:** 10.3389/fneur.2025.1714325

**Published:** 2025-12-18

**Authors:** Yifan Zhang, Cheng Cao, Xin Huang, Xinmin Zhou

**Affiliations:** 1 The Jiangyin Clinical College of Xuzhou Medical University, Jiangyin, China; 2 Neurosurgery Department, Jiangyin People's Hospital, Jiangyin, China

**Keywords:** tandem lesions, endovascular procedures, prognosis, mechanical thrombectomy, acute ischemic stroke

In the published article, Xinmin Zhou was erroneously assigned affiliation 2 “Neurosurgery Department, Jiangyin People's Hospital, Jiangyin, China”. The correct affiliation is 1 “The Jiangyin Clinical College of Xuzhou Medical University, Jiangyin, China.”

The order of the authors was also corrected from: Yifan Zhang^*^, Xinmin Zhou^*^, Cheng Cao and Xin Huang to: Yifan Zhang^*^, Cheng Cao, Xin Huang and Xinmin Zhou^*^.

The original article has been updated.

