# Supplementary material for: Analysis of risk factors for poor prognosis after endovascular treatment of tandem lesions in acute ischemic stroke
Source: Front Neurol. 2025 Sep 18;16:1628374. doi: 10.3389/fneur.2025.1628374 (PMC12490274; doi:10.3389/fneur.2025.1628374)
Supplement: Supplementary file 1 [file Table_1.docx]

Table S1 Univariable logistic regression results for poor 90-day outcome

| **Variable** | **OR (95% CI)** | **P value** | **Adusted P value** |
| --- | --- | --- | --- |
| Age | 1.05 (1.01–1.10) | 0.13 | 0.069 |
| Sex | 2.06 (0.64–6.96) | 0.230 | 0.400 |
| Hypertension | 1.77 (0.67–4.73) | 0.250 | 0.400 |
| Diabetes | 1.23 (0.37–4.48) | 0.736 | 0.736 |
| CAD | 3.18 (0.44–63.90) | 0.312 | 0.416 |
| Smoking | 1.23 (0.37–4.48) | 0.736 | 0.736 |
| AF | 1.57 (0.38–7.93) | 0.549 | 0.671 |
| anterior_TL | 0.48 (0.14–1.47) | 0.214 | 0.400 |
| recanal_time | 1.00 (0.99–1.01) | 0.587 | 0.671 |
| nihss | 1.15 (1.06–1.27) | 0.002 | 0.032 |
| stent | 2.34 (0.77–8.09) | 0.149 | 0.389 |
| mtici | 0.11 (0.01–0.60) | 0.038 | 0.152 |
| neutrophil | 1.48 (1.13–2.00) | 0.007 | 0.056 |
| lymphocyte | 3.48 (0.65–22.29) | 0.162 | 0.389 |
| platelet | 1.00 (0.99–1.00) | 0.278 | 0.404 |
| monocyte | 0.04 (0.00–3.43) | 0.170 | 0.389 |
